# Impact of metformin use among tuberculosis close contacts with diabetes mellitus in a nationwide cohort study

**DOI:** 10.1186/s12879-019-4577-z

**Published:** 2019-11-06

**Authors:** Ming-Chia Lee, Chih-Hsin Lee, Meng-Rui Lee, Jann-Yuan Wang, Shih-Ming Chen

**Affiliations:** 1 Department of Pharmacy, New Taipei City Hospital, #3, Sec. 1, New Taipei Blvd., Sanchong Dist, New Taipei City, 24141 Taiwan; 2 School of Pharmacy, College of Pharmacy, Taipei Medical University, #250, Wuxing St, Taipei, 11031 Taiwan; 3 Division of Pulmonary Medicine, Department of Internal Medicine, Wan Fang Hospital, Taipei Medical University, #111, Sec. 3, Xinglong Rd., Wenshan Dist, Taipei, 11696 Taiwan; 4 Division of Pulmonary Medicine, Department of Internal Medicine, School of Medicine, College of Medicine, Taipei Medical University, #250, Wuxing St, Taipei, 11031 Taiwan; 5 Department of Internal Medicine, National Taiwan University Hospital, Hsin-Chu, Branch, #25,Lane 442,Sec.1,Jingguo Rd., Hsin-chu, 300 Taiwan; 6 Department of Internal Medicine, National Taiwan University Hospital, #7, Chung-Shan South Road, Taipei, 10002 Taiwan; 7 Institute of Epidemiology and Preventive Medicine, College of Public Health, National Taiwan University, #17, Xuzhou Rd., Zhongzheng Dist, Taipei, 10055 Taiwan

**Keywords:** Close contact, Diabetes mellitus, Host-directed therapy, Metformin, Tuberculosis

## Abstract

**Background:**

The protective effect of metformin against active tuberculosis (TB) among TB close contacts is unknown.

**Methods:**

TB close contacts with diabetes mellitus (DM) and normal renal function were selected from the National Health Insurance Research Database of Taiwan. Metformin users were patients who received ≥90 cumulative defined daily doses within 1 year before the index date. For each metformin user, a propensity-score matched metformin nonuser and an age- and sex-matched healthy TB close contact were selected. The outcome was incident TB, identified using previously validated diagnostic criteria. Independent predictors were investigated using stratified Cox regression analysis. Interaction analysis was also performed.

**Results:**

A total of 5846 TB close contacts who were metformin users, metformin non-users, and healthy contacts were analysed. The incidence of active TB was 755 (600–938), 1117 (927–1335), and 526 (393–689) cases per 100,000 person-years in each group, respectively. Multivariate analysis revealed that healthy contacts had the lowest risk of developing active TB (adjusted hazard ratio [aHR]: 0.42 [0.30–0.60]) and metformin use partially reversed the risk associated with DM (aHR: 0.73 [0.54–0.98]). Subpopulation analysis revealed a significant interaction between insulin use and metformin use.

**Conclusions:**

Metformin use is associated with a lower risk of developing active TB among TB close contacts with DM, especially for insulin users. It may be an alternative choice for primary prevention against active TB if no contraindications exist. However, prospective studies are needed to confirm the findings.

## Background

Tuberculosis (TB) is one of the most lethal infectious diseases worldwide. In 2016, 6.3 million new cases of TB and 1.3 million TB-related deaths were reported [1]. Thus, curing existing cases of TB, preventing new infections with *Mycobacterium tuberculosis* (MTB), and interrupting progression to active disease are critical. Since TB contact is an important risk factor for TB development [2, 3] and latent tuberculosis infection (LTBI) cases have a 10% life-time risk of progression to active TB [4], TB contact investigation is therefore an essential component of TB control policies, aimed at early detection and prompt treatment of infected patients [5].

Diabetes mellitus (DM) is a common systemic co-morbidity of TB patients, and significantly increases the risk of developing TB (adjusted hazard ratio [aHR]: 1.29 [1.15–1.45]) [6]. The World Health Organization has indicated that globally, 422 million adults had DM in 2014, a number nearly double that of 1980 [7]. By enhancing the host cell response to the pathogen [8], metformin has been reported as a host-directed therapy for TB [9]. Some clinical studies have shown that metformin is associated with a reduced incidence of TB [9–11], time-to-culture conversion [12], relapse rate [13], mortality [14], and protection against LTBI [9]. Studies are lacking to address this issue in TB close contacts, a well-known group at high risk of developing active TB and also a recommended target population for LTBI interventions. It remains unknown whether or not use of metformin can aid in TB prevention among this high-risk, yet relatively healthy and young population. Nevertheless, understanding TB epidemiology in this special DM population should aid in policy making and resources allocation in LTBI programs.

TB contact investigation is an essential part of the TB control program in Taiwan [15]. The completion rate of TB contact investigation has reached > 95%. TB contact investigation in Taiwan, therefore, serves as a valuable and unbiased resource for research, never previously used to address the protective effect of metformin against active TB. Therefore, we conducted this cohort study of TB close contacts to investigate the potential protective effect of metformin against active TB disease.

## Methods

### Ethics statement

This study was approved by the Institutional Review Board of National Taiwan University Hospital (No.: 201606086RINA) and the requirement for informed consent was waved because this retrospective study used encrypted data and did not add any risk to the participants.

### TB contact investigation in Taiwan

A summary of the public health policy on TB contact investigation in Taiwan during the study period is illustrated in Supplementary E-Table 1 [16]. The target population for contact investigation included the following: 1) people living with an index case; 2) people who had contact with the index case for > 8 h per day or > 40 h within the overall infectious period (close contact); or 3) other specified conditions that warrant investigation (i.e., outbreak or cluster cases of TB in schools or congregate settings). Briefly, TB contact investigation during the study period required that a chest X-ray (CXR) be completed for contacts within 1 month after diagnosis of the index case. For contacts of an index case who had an acid-fast smear (AFS)-positive sputum sample, positive sputum culture, or cavitation on CXR, a CXR was repeated at 1 year after the diagnosis of the index case, regardless of LTBI treatment.

**Table 1:** Clinical characteristics of metformin users stratified by dose, propensity score-matched non-users, and a non-user matched healthy cohort

Characteristics	Healthy cohorts (*n* = 5846)	Metformin nonusers (*n* = 5846)	*p*-value*	Metformin users			
				All (n = 5846)	*p*-value^#^	Low cumulative exposure (*n* = 5587)	High cumulative exposure (*n* = 259)
Age at index date							
< 45	570 (9.8%)	570 (9.8%)		531 (9.1%)		506 (9.1%)	25 (9.7%)
45 to 65	3030 (51.8%)	3028 (51.8%)		3067 (52.5%)		2916 (52.2%)	151 (58.3%)
≥ 65	2246 (38.4%)	2248 (38.5%)		2248 (38.5%)		2165 (38.8%)	83 (32.0%)
Sex					>		
Male	2464 (42.1%)	2464 (42.1%)		2464 (42.1%)		2360 (42.2%)	104 (40.2%)
Female	3382 (57.9%)	3382 (57.9%)		3382 (57.9%)		3227 (57.8%)	155 (59.8%)
aDCSI score							
≥ 3	0 (0%)	1992 (34.1%)		2010 (34.4%)		1917 (34.3%)	93 (35.9%)
1 to 2	0 (0%)	2312 (39.5%)		2294 (39.2%)		2187 (39.1%)	107 (41.3%)
0	100 (0%)	1542 (26.4%)		1542 (26.4%)		1483 (26.5%)	59 (22.8%)
Type 1 DM	0 (0%)	87 (1.5%)	–	87 (1.5%)		80 (1.4%)	7 (2.7%)
Liver cirrhosis	0 (0%)	23 (0.4%)	–	23 (0.4%)		22 (0.4%)	1 (0.4%)
Coexisting medical condition							
Previous TB history	76 (1.3%)	384 (6.6%)	< 0.001	224 (3.8%)	< 0.001	220 (3.9%)	4 (1.5%)
COPD	0 (0%)	487 (8.3%)	–	352 (6.0%)	< 0.001	344 (6.2%)	8 (3.1%)
Malignancy	0 (0%)	269 (4.6%)	–	234 (4.0%)	0.115	222 (4.0%)	12 (4.6%)
Bronchiectasis	0 (0%)	115 (2.0%)	–	64 (1.1%)	< 0.001	60 (1.1%)	4 (1.5%)
Transplantation	0 (0%)	2 (0.03%)	–	5 (0.09%)	0.453	5 (0.1%)	0 (0%)
HIV/AIDS	0 (0%)	2 (0.03%)	–	2 (0.03%)	> 0.999	2 (0.04%)	0 (0%)
Other co-morbidity^$^	0 (0%)	105 (1.8%)	–	96 (1.6%)	0.569	92 (1.6%)	4 (1.5%)
Urban contact area	4084 (69.9%)	3973 (68.0%)	0.027	4035 (69.0%)	0.223	3842 (68.8%)	193 (74.5%)
Local TB incidence (/100,000 PYs)	58.5 ± 16.1	59.7 ± 15.7	0.888	59.1 ± 15.7	0.035	59.1 ± 15.8	59.2 ± 14.98
Low income	391 (6.7%)	410 (7.0%)	0.487	426 (7.3%)	0.591	408 (7.3%)	18 (6.9%)
Medical visits in 3 months	2.4 ± 2.5	3.8 ± 3.2	< 0.001	4.0 ± 2.9	< 0.001	4.0 ± 2.9	4.0 ± 2.6
Statin users	0 (0%)	1108 (19.0%)	–	1559 (26.7%)	< 0.001	1482 (26.5%)	77 (29.7%)
Corticosteroid users	0 (0%)	80 (1.4%)	–	67 (1.1%)	0.322	64 (1.1%)	3 (1.2%)
Insulin users	0 (0%)	2284 (39.1%)	–	2399 (41.1%)	< 0.001	2278 (40.8%)	121 (46.7%)
Other OHA users	0 (0%)	5211 (89.1%)	–	5551 (95.0%)	< 0.001	5295 (94.8%)	256 (98.8%)
Latent TB infection	256 (4.4%)	391 (6.7%)	< 0.001	200 (3.4%)	< 0.001	185 (3.3%)	15 (5.8%)
IPT	67 (1.1%)	94 (1.6%)	0.032	65 (1.1%)	0.026	60 (1.1%)	5 (1.9%)
Follow-up duration (days)	582.4 ± 226.6	648.7 ± 177.9	< 0.001	637.3 ± 166.1	< 0.001	635.1 ± 167.5	685.7 ± 121.9
Incident TB events	49 (0.8%)	116 (2.0%)	< 0.001	77 (1.3%)	0.006	74 (1.3%)	3 (1.2%)

Abbreviations: aDCSI, adapted Diabetes Complications Severity Index; HIV/AIDS, human immunodeficiency virus/acquired immunodeficiency syndrome; COPD, chronic obstructive pulmonary disease; DM, diabetes mellitus; IPT, early adherent isoniazid preventive therapy; OHA, oral hypoglycemic agents; PY, person-years; TB, tuberculosis

Data are expressed as the number (%) unless otherwise specified

* *p* value of healthy cohorts vs. metformin nonusers and ^#^ *p* value of metformin users vs. nonusers in paired *t* test for continuous variables and McNemar test for categorical variables

^$^ Including pneumoconiosis, psoriasis, rheumatoid arthritis, and ankylosing spondylitis

Before 2016, only tuberculin skin test (TST) was used to diagnose LTBI during contact investigation under the National TB Program in Taiwan. The LTBI program in Taiwan covered close contacts younger than 13 years old since 2008, and was expanded to covered close contacts older than 13 years old and those born after 1986. Therefore, LTBI testing was not universally performed in all TB close contacts. Even if close contacts were screen-positive, preventive therapy was not obligatory (not regulated by law). Furthermore, to be reimbursed, doctors needed to report every newly-diagnosed TB case [16].

### Study setting, data source, and cohort participants

In 2005, Taiwan is a country with an intermediate TB burden, with an incidence of TB was 72.5 per 100,000 population; this decreased gradually to 45.7 per 100,000 population in 2015 [17]. This population-based cohort study was conducted utilizing the National Health Insurance Research Database (NHIRD) of Taiwan [3]. National Health Insurance (NHI) is the mandatory single-payer health insurance program launched on 1 March, 1995, and provides medical care coverage for 99% of the > 22 million residents of Taiwan. The database of the NHI program contains registration files and original claims data for reimbursement. The NHIRD was originally derived from the NHI system by the NIH Administration (the former Bureau of National Health Insurance), Ministry of Health and Welfare (the former Department of Health) of Taiwan, and was maintained for research purposed by the National Health Research Institutes, Taiwan. In the field of mycobacteriology, NHIRD has served as an important platform for epidemiologic studies [3, 11, 18].

From 2008 to 2013, cohort participants (TB close contacts) were selected based on diagnosis (9th revision of the International Classification of Diseases, clinical modification [ICD-9-CM] V01.1 or 795.5) and medical payment (co-payment exemption, as part of the National TB Program of Taiwan) according to a previous publication [3]. The date of the visit mentioned above was set as the index date. We excluded those participants with active TB and those receiving treatment before the index date. Contacts were followed until occurrence of the outcome, which was defined as the development of active TB disease within 2 years after the index date or 31 December 2013.

### Definition of DM

We used the methodology previously described by the previous study [19]. The DM cohort included patients who had at least one hospital admission or at least three outpatient visits with a DM diagnostic code (ICD-9-CM code: 250) within 365 calendar days before the index date. In this study, patients were considered DM patients if they received treatment with insulin or diabetes-specific hypoglycemic agents (see Supplemental file) for ≥90 cumulative defined daily doses (DDDs) [20] within 365 days [19]. The onset of DM was defined as the first date of anti-DM medication administration.

DM patients were excluded for the following reasons: 1) onset of DM within 365 days before the index date; or 2) diabetes visit claims within 270 days prior to parturition (to exclude women with gestational diabetes). Also DM patients with a diagnosis of end-stage renal disease (ESRD) or chronic kidney disease (CKD) before the index date were also excluded to avoid confounding by indication. Type 1 DM was noted according to previous publications [19].

### Exposure of interest: use of metformin

Metformin users were defined as those with ≥90 cumulative DDDs of total prescriptions for metformin within 1 year prior to the index date. One propensity score (PS)-matched metformin non-user was selected for each metformin-using DM patient. PS matching was performed to estimate and control for the probability of receiving metformin through a multivariate logistic regression model. The variables included age at index date (< 65 or ≥ 65 years), sex, the adapted Diabetes Complications Severity Index (aDCSI) score (0 or ≥ 1), type 1 DM, and liver cirrhosis. The aDCSI score has been validated in claims data and is a good measure of diabetes severity [21]. For each metformin non-user, a healthy contact was selected by using 1:1 case-matching for sex and age at index date. A healthy contact was defined as a TB close contact without DM, chronic obstructive pulmonary disease (COPD), bronchiectasis, ESRD, CKD, diabetic nephropathy, hypertensive nephropathy, liver cirrhosis, malignancy, pneumoconiosis, rheumatologic disease (including ankylosing spondylitis, rheumatoid arthritis, psoriasis), transplant or acquired immunodeficiency syndrome (AIDS).

### Outcome of interest: incident TB

Newly-diagnosed TB within 2 years after the index date was considered the outcome of interest. The diagnosis of TB was established by considering both the diagnosis code and the prescription of anti-TB drugs, as described in a previous report [18]. The diagnostic criteria for TB had been validated by examining patients suspected to have TB based on an AFS-positive sputum sample and mycobacterial culture at a medical center in northern Taiwan, with a sensitivity of 99.13% and specificity of 99.90% [20].

### Possible confounding factors

The confounding factors that may affect the risk of TB were recorded at the index date and included age, sex, co-morbidity, medical visits within 3 months prior to the index date, TB incidence in the contact area, urban contact area, economic status, underlying diseases, low income (an annual household income < 4500 US dollars), LTBI, early adherent isoniazid preventive therapy (IPT), use of statins, insulin use, use of oral hypoglycemic agents (OHAs) other than metformin, and corticosteroid use (see Supplemental file for definitions of confounding factors and drug codes) [18, 22].

Medication use was identified on the basis of prescriptions in each category for ≥70 cumulative DDDs of prednisolone and 90 cumulative DDDs for a statin, insulin and OHAs within 1 year before the index date. Contact area was defined as the area in which the health care institute was located at the index date. A contact area was considered to be urban if the population density of the area (adapted from the Taiwan Ministry of the Interior) exceeded 1500 persons/km2. The incidence in a high contact area was defined as the incidence of TB (adapted from the Centers for Disease Control, Taiwan [23]) > 100 per 100,000 person-years. Medical visits within 3 months prior to the index date were defined as the sum of medical visits (each outpatient department visit was equal to 0.5, with a maximum of 1 per day; each day of hospitalization was equal to 1). IPT was defined as starting isoniazid within 180 days after the index date and take it for at least 180 days. LTBI was defined as a compatible diagnosis with the ICD-9-CM code of 795.5 or a prescription for isoniazid within 6 months after the index date.

### Statistical analysis

The data are indicated as the mean ± standard deviation or number (%), as appropriate. The McNemar test and a paired t-test for matched samples were used to evaluate the intergroup differences for categorical and continuous variables. Kaplan–Meier curves for time-to-incident TB were generated and compared using the log-rank test. The independent factors associated with incident TB, after adjusting for all above-mentioned confounders were identified by stratified multivariable Cox proportional hazard regression analysis.

For the dose-response analysis, we divided the cumulative exposure to metformin within 1 year before the index date into low [90 ≤ cumulative DDDs < 360], and high cumulative exposure [cumulative DDDs ≥360]. Interaction analysis was performed between metformin and age, sex, aDCSI score, type 2 DM, use of insulin and use of OHAs other than metformin. Results were considered significant when a two-sided *p*-value was less than 0.05. All analyses were performed using the Statistical Product and Service Solutions Version 18.0 (IBM SPSS Statistics for Windows, Armonk, NY, USA).

## Results

### Case selection and clinical characteristics

**Fig. 1:**
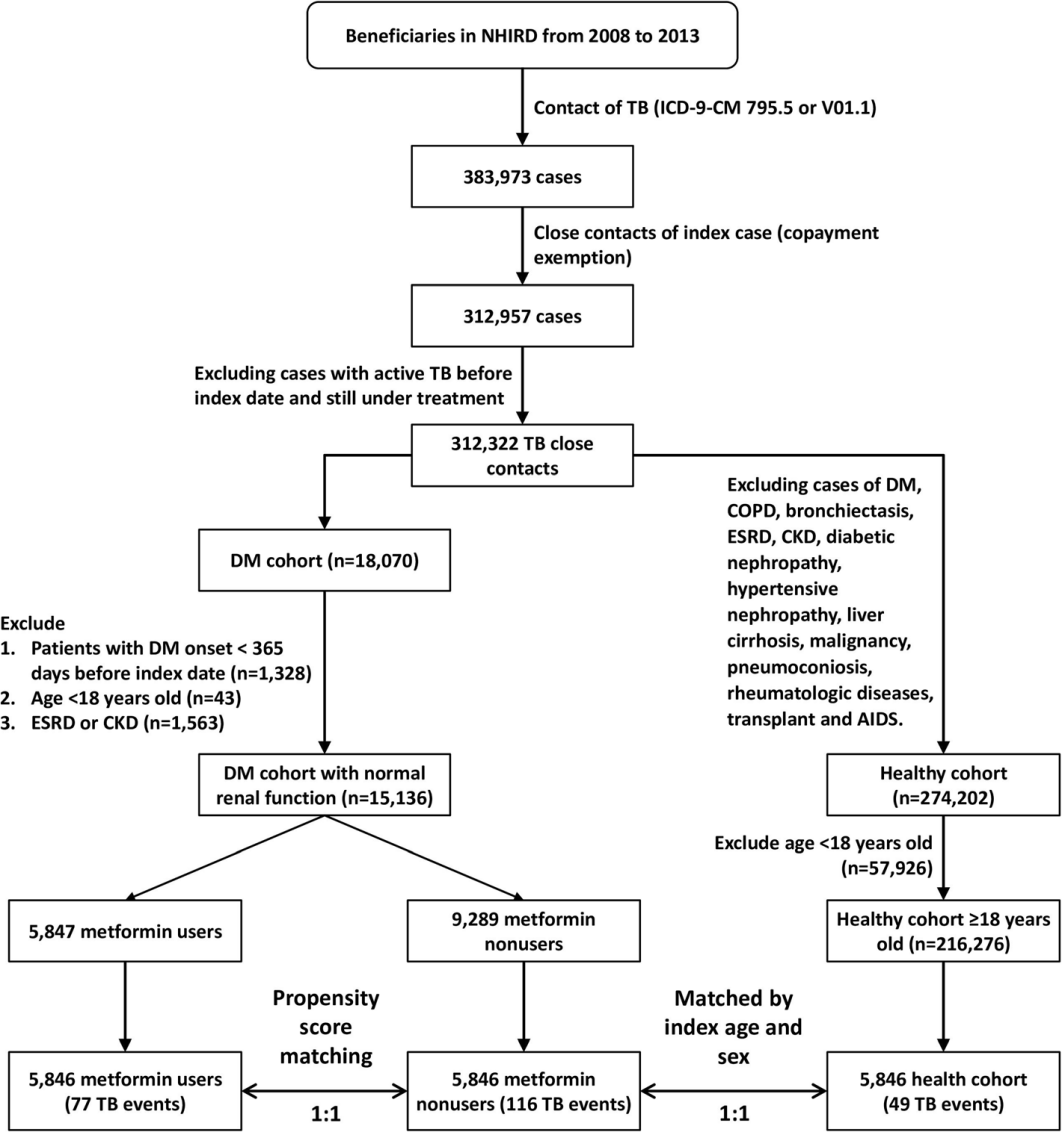
Flowchart of study design and case selection (AIDS: acquired immunodeficiency syndrome; CKD: chronic kidney disease; COPD: chronic obstructive pulmonary disease; DM: diabetes mellitus; ESRD: end-stage renal disease; ICD-9-CM: International Classification of Diseases, ninth revision, clinical modification; NHIRD: National Health Insurance Research Database; TB: tuberculosis)

Between 2008 and 2013, a total of 383,973 of TB close contacts were identified (Fig. 1). Among these, 18,070 had DM and 15,136 had DM with normal renal function. Of the TB close contacts with DM, 5847 were classified as metformin users. After matching, 5846 metformin users, 5846 metformin non-users, and 5846 healthy TB close contacts > 18 years of age were selected for further study. The logistic regression model for propensity-score matching is shown in E-Table 2. Before propensity score matching, the distribution of age between metformin users and non-users was significantly different (E-Table 3). The age, sex, aDCSI score, prevalence of type 1 DM and prevalence of liver cirrhosis were similar in metformin users and nonusers (*p* > 0.999) (Table 1). Compared with the metformin nonusers, metformin users had a lower prevalence of co-morbidities, including a TB history, COPD, and bronchiectasis. Among the three groups, < 0.1% had human immunodeficiency virus/AIDS and there was no difference in the proportion of cases with low income. Metformin users had the most medical visits in three months (4.0 ± 2.9) among the three groups. The proportion of corticosteroid users was similar between the metformin users and non-users, whereas the former group contained a significantly higher proportion of statin users (26.7% vs. 19.0%, *p* < 0.001). The proportion of insulin users (41.1% vs. 39.1%, p < 0.001) and other OHA users (95.0% vs. 89.1%, p < 0.001) was higher in metformin users than in non-users. Metformin non-users had the highest prevalence of LTBI in the three groups and were more likely to receive IPT than were metformin users (1.6% vs. 1.1%, *p* = 0.026). The cumulative DDDs of metformin users were still higher than those of non-users in both the first and second year after the index date (E-Table 4).

**Table 2:** Independent Predictors of tuberculosis development among tuberculosis close contact cohort by stratified multivariable Cox proportional hazard regression analysis

Variables	adjusted Hazard ratio (95% CI)	*p*-value
DM and metformin status		
DM, metformin nonusers	Reference	
DM, metformin users	0.73 (0.54–0.98)	0.035
Healthy cohort	0.42 (0.30–0.60)	< 0.001
Statin users	0.58 (0.35–0.97)	0.038
Bronchiectasis	9.62 (1.09–84.81)	0.041

Abbreviations: DM, diabetes mellitus

Adjusted variables included age, male, adapted Diabetes Complications Severity Index, previous tuberculosis history, urban contact area, local TB incidence (/100,000 person-years), low income, medical visits in 3 months, statin users, type 1 diabetes mellitus, chronic obstructive pulmonary disease, liver cirrhosis, transplantation, acquired immunodeficiency syndrome, bronchiectasis, early adherent isoniazid preventive therapy, latent tuberculosis infection, use of statins, corticosteroids, insulin, and oral hypoglycemic agents other than metformin, malignancy, and other co-morbidities (pneumoconiosis, psoriasis, rheumatoid arthritis, and ankylosing spondylitis).

### Incident TB cases in the NHIRD

**Fig. 2:**
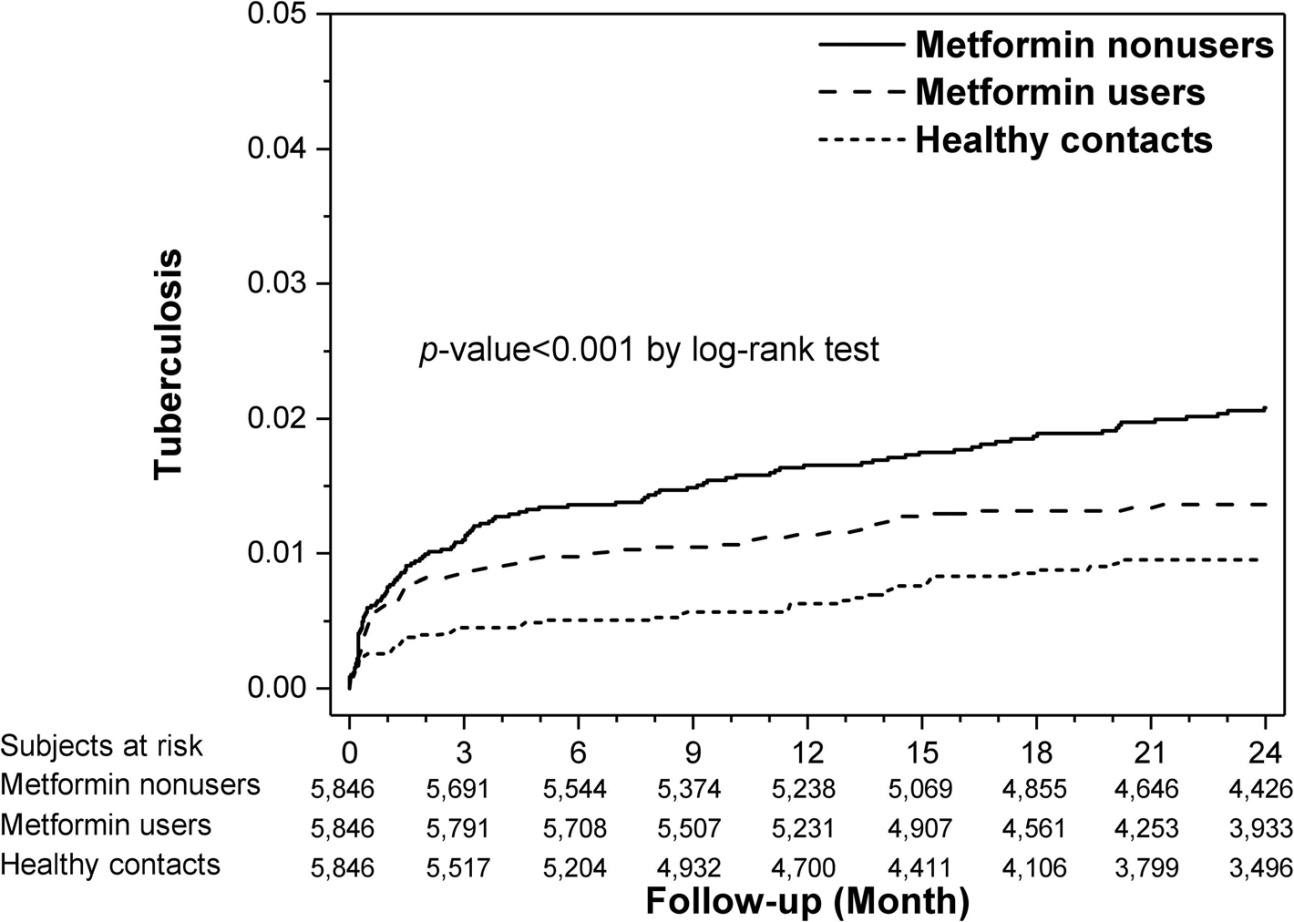
Kaplan–Meier curves depicting time-to-active tuberculosis among healthy contacts, metformin users, and non-users

The follow-up duration was 582.4 ± 226.6 days in healthy contacts, 648.7 ± 177.9 days in metformin non-users, and 637.3 ± 166.1 days in metformin users. During follow-up, a total of 242 cases (49 healthy contacts, 77 metformin users, and 116 metformin non-users) developed active TB, corresponding to 809 (95% CI 712–916) per 100,000 person-years (526 [393–689], 755 [600–938] and 1117 [927–1335] cases per 100,000 person-years in healthy contacts, metformin users, and metformin non-users, respectively). Time-to-TB significantly differed among the three groups (*p* = 0.001 by log-rank test; Fig. 2).

Within 2 months after the index date, metformin non-users had significantly more incident TB cases than metformin users or healthy contacts (58 [1.0%] vs. 48 [0.8%] vs. 23 [0.4%], respectively; *p* < 0.001 by chi-square test).

### Risk factors for TB in DM patients with TB close contact

Compared to metformin non-users, healthy contacts had a lower risk of developing active TB (crude hazard ratio [cHR]: 0.44 [0.32–0.62]), as did metformin users (cHR: 0.69 [0.51–0.92]; E-Table 5). Adjusting for LTBI status did not change the HR of developing active TB between metformin users and nonusers. The cHRs of other factors are also shown in E-Table 5.

Stratified multivariable Cox proportional hazard regression analysis revealed that compared to metformin nonusers, healthy contacts had a lower risk of developing active TB (aHR: 0.42 [0.30–0.60]), as did metformin users (aHR: 0.73 [0.54–0.98]; Table 2).

### Sub-population analysis

**Fig. 3:**
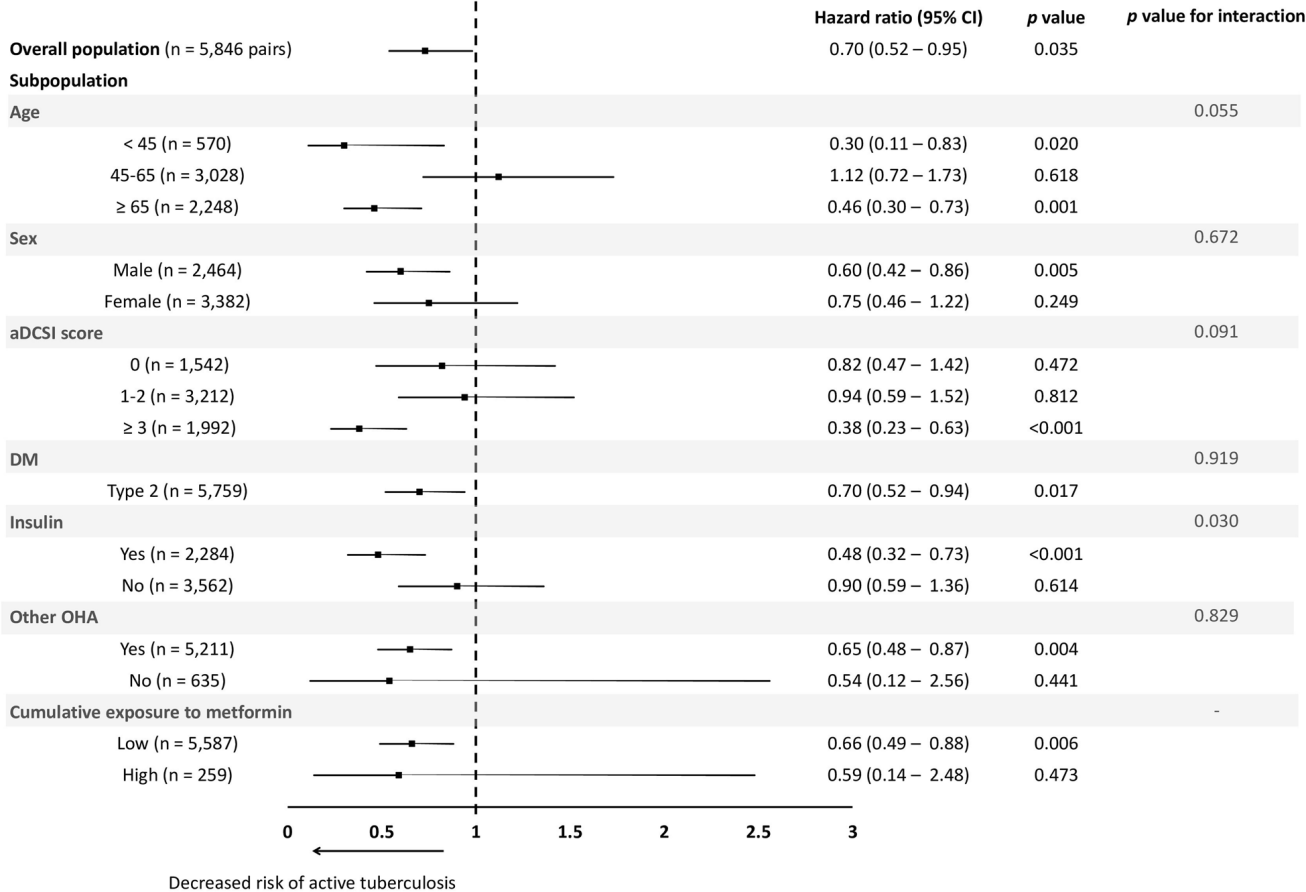
Forest plot showing the adjusted hazard ratio of metformin use on the development of active tuberculosis in overall population and different subgroups by multivariable Cox proportional hazard regression analysis

Sub-population analysis (Fig. 3) revealed that metformin was protective in DM patients < 45 years (aHR: 0.30 [0.11–0.83]), those ≥65 years (aHR: 0.46 [0.30–0.73]), males (aHR: 0.60 [0.42–0.86]), those having an aDCSI score ≥ 3 (aHR: 0.38 [0.23–0.63]), type 2 DM patients (aHR: 0.70 [0.52–0.94]), those using insulin (aHR: 0.48 [0.32–0.73]), and those using other OHAs (aHR: 0.65 [0.48–0.87]). Only the interaction between insulin and metformin use was statistically significant (*p* < 0.030) (Fig. 3). In the dose–response analysis for metformin use, the point estimate of the aHR shows a trend of decreasing as the dose of metformin increased, from 0.66 [0.49–0.88] for low cumulative exposure to metformin to 0.59 [0.14–2.48] for high cumulative exposure.

## Discussion

This is the first study of TB close contacts and indicates that metformin protects DM patients from developing active TB. There were two major findings. First, among TB close contacts, receiving metformin for ≥90 cumulative DDDs within 1 year partially reversed the increased risk of active TB associated with DM. Second, metformin use was associated with a lower risk of TB especially among insulin users.

Singhal et al. [9] reported that metformin serves as an adjunct anti-TB therapy by reducing the intracellular growth of MTB. In the first two human studies, the association between metformin use and a lower TB risk was not adjusted by controlling for the potential confounding effect of impaired renal function [9, 10], the most common concern for metformin use [24]. Indeed, patients with chronic renal failure and on dialysis had a 3.62-fold increased risk of TB, compared with the general population [25]. Consequently, the advantages of metformin use in the two studies are likely to be confounded by indication, rather than reflecting a true protective effect.

One interesting findings in this study is that fewer metformin users had LTBI than non-users, which is similar to the finding of Singhal et al. [9]. Magee et al. also reported lower prevalence of LTBI among DM patients with metformin and statin use than in those without (TST-positive rate: 4% vs. 10%) [26]. Furthermore, fewer metformin users than non-users developed incident TB within two months after the index date. Taken together, these findings imply that metformin may provide a TB-protecting effect that is independent of LTBI treatment. However, because LTBI testing and treatment were not universally performed in all study subjects, further research is needed to confirm these findings.

In the cohort study of Pan et al. [11], newly-diagnosed DM patients were identified from the Longitudinal Health Insurance Database of Taiwan, which is composed of the claim data of 1,000,000 randomly sampled beneficiaries and those with CKD were excluded. Specifically, a 66% reduction in TB risk among metformin users was shown compared to sulfonylurea users (aHR 0.34 [0.17–0.67]) [11]. Similarly, in the current study subjects with CKD were also excluded. In addition, high cumulative exposure to metformin tended to be more protective, though not significant, than low cumulative exposure, probably due to the small number in the former group. In addition, the protective effect of metformin was less in the current study, resulting in only a 30% risk reduction among TB close contacts with DM, but not CKD. The reasons for the discrepancy between the two studies might be the differences in the target populations. Pan et al. selected newly-diagnosed DM patients between 2003 and 2013 from the Longitudinal Health Insurance Database. In the current study, TB contacts with DM diagnosed between 2008 and 2013 were selected from the whole NHIRD (approximately 25,000,000 individuals). Therefore, up to an estimated 4% (1,000,000/25,000,000) of our study subjects were also enrolled in Pan’s study, not considering the other differences in inclusion and exclusion criteria between the two studies.

The relative risk of active TB disease between DM patients and healthy TB close contacts (aHR: 4.04 [1.51–10.74]) [27] was higher than that between DM patients and healthy subjects in the general population (aHR 3.11 [2.27–4.26]) [28]. Furthermore, the incidence of active TB disease in TB close contacts with DM in this study was 809 (95% CI 712–916) per 100,000 person-years, which was 2.64 to 12.1 fold higher than that of DM patients in the general population (66.7–306/100,000 person-years) [28]. Further studies are needed to confirm whether or not the benefit of metformin use wanes as the risk of active TB disease increases. Based on stratified multivariable Cox proportional hazards regression, statin use significantly decreased the risk of active TB and corticosteroid use significantly increased that risk, results similar to those found in previous studies [29, 30].

In subgroup analysis revealed that metformin was associated with a lower risk of TB, especially in insulin users. Insulin is the drug of choice for DM patients with poor glucose control [31]; therefore, insulin use may be a surrogate of poorly controlled DM. Metformin was also associated with a lower risk of TB among DM patients with a higher aDCSI score (≥3), although the interaction between metformin and aDCSI was not significant. We speculate that metformin might be more protective as the severity of DM increases. However, more studies are needed to confirm this finding.

This study had several limitations. First, the NHIRD does not include data on fasting blood glucose or hemoglobin A1c levels of information on body habitus and life-style. Therefore, many important confounders, such as quality of glycemic control, severity of DM, underweight, and smoking, were not measurable. Second, most of the study subjects received no LTBI testing or preventive therapy. Therefore, we could not precisely analyze the impact of these factors on the preventive effect of metformin. Finally, because this is a retrospective analysis using a claims database, the causal effect of metformin in reducing the risk of TB cannot be inferred.

## Conclusions

The findings of this nationwide cohort study on TB close contacts suggest that metformin use is associated with a lower TB risk among DM patients, especially for insulin users. Metformin may be an alternative choice for primary prevention against active TB in those with close contact if no contraindications exist. However, prospective studies are needed to confirm the findings.

## Supplementary information

**Additional file 1.** Supplemental methods and results. This file includes definitions of co-morbidities, list of drugs and anatomic therapeutic chemical codes, E-Table S1, E-Table S2, E-Table S3, E-Table S4 and E-Table S5.

## Data Availability

The original datasets used and analyzed during the current study are closed for releasing now. The original databases used and/or analysed during the current study, which were released and permitted by the National Health Research Institutes, are closed for releasing now. The working datasets for statistical analysis in the current study are available from the corresponding author on reasonable request.

## Abbreviations

aDCSI: adapted Diabetes Complications Severity Index
AFS: acid-fast smear
aHR: adjusted hazard ratio
AIDS: Acquired immunodeficiency syndrome
cHR: crude hazard ratio
CKD: chronic kidney disease
COPD: chronic obstructive pulmonary disease
CXR: chest X-ray
DDD: defined daily dose
DM: diabetes mellitus
ESRD: end-stage renal disease
ICD-9-CM: International Classification of Diseases, ninth revision, clinical modification
IPT: isoniazid preventive therapy
LTBI: latent tuberculosis infection
MTB: *Mycobacterium tuberculosis*
NHI: National Health Insurance
NHIRD: National Health Insurance Research Database
OHA: oral hypoglycemic agents
PS: propensity-score
TB: tuberculosis
TST: tuberculin skin test
