# Supplementary material for: Impact of metformin use among tuberculosis close contacts with diabetes mellitus in a nationwide cohort study
Source: BMC Infect Dis. 2019 Nov 6;19:936. doi: 10.1186/s12879-019-4577-z (PMC6836500; doi:10.1186/s12879-019-4577-z)
Supplement: Supplementary file 1 — Additional file 1. Supplemental methods and results. This file includes definitions of co-morbidities, list of drugs and anatomic therapeutic chemical codes, E-Table S1, E-Table S2, E-Table S3, E-Table S4 and E-Table S5. [file 12879_2019_4577_MOESM1_ESM.docx]

**Supplemental Methods and Results**

**Definitions of Co-morbidities**

**Chronic obstructive pulmonary disease (COPD)**: at least two outpatient or inpatient records with compatible diagnoses (ICD-9-CM codes 491, 492, or 496) and a prescription for at least two COPD-specific medications or one COPD-specific medication plus at least one airway medication in 90 days. (The COPD-specific medications include corticosteroids [inhaled, oral, or parenteral], beta2-agonists [long- or short-acting; inhaled or oral], anticholinergics [ipratropium or tiotropium], aminophylline, and theophylline. Airway medications included oral antitussives, mucolytic agents, and sympathomimetics.).

**Malignancy:**

**Pulmonary cancer**: compatible ICD-9-CM code (162) from the Registry for Catastrophic Illness Patient Database (RCIPD), which is a separate file section of the National Health Insurance Database and requires histologic, radiologic, or laboratory confirmation of the disease.

**Extra-pulmonary cancer:** ICD-9-CM codes 140–208, but not 162, from the RCIPD.

**Liver cirrhosis:** ICD-9-CM code 571 in the RCIPD.

**Human immunodeficiency virus/Acquired immunodeficiency syndrome**: two or more records with compatible diagnoses (ICD-9-CM codes 042 and V08) and prescription for highly-active antiretroviral therapy in 180 days.

**Pneumoconiosis**: ICD-9-CM codes 500–505 in the RCIPD.

**Bronchiectasis**: At least two outpatient records or one inpatient record with ICD-9-CM code 494 within 90 days.

**Rheumatoid arthritis**: At least three outpatient records with ICD-9-CM code 714 entered by a rheumatologist or dermatologist within 180 days or one inpatient record with ICD-9-CM code 714.

**Ankylosing spondylitis**: At least three outpatient records with ICD-9-CM code 720 entered by a rheumatologist or dermatologist within 180 days or one inpatient record of ICD-9-CM code 720.

**Psoriasis**: At least three outpatient records with ICD-9-CM code 696 entered by a rheumatologist or dermatologist within 180 days or one inpatient record with ICD-9-CM code 696.

**End-stage renal disease and chronic kidney disease:** compatible diagnoses (ICD-9-CM code 585) in at least two outpatient records, at least one inpatient record, or in the RCIPD.

**List of Drugs and Anatomic Therapeutic Chemical (ATC) Codes**

**Diabetes-specific hypoglycemic agents:** acarbose, acetohexamide, buformin, chlorpropamide, glibornuride, gliclazide, glimepiride, glipizide, gliquidone, glyburide, insulin, metformin, nateglinide, pioglitazone, repaglinide, rosiglitazone, tolazamide, and tolbutamide (ATC codes: A10BA, A10BB, A10BD, A10BF, A10BG, A10BH, and A10BX)

**Anti-tuberculosis drugs:** isoniazid, rifampin, rifabutin, ethambutol, pyrazinamide, prothionamide, terizidone, streptomycin, kanamycin, quinolones, cycloserine, and aminosalicylic acid (ATC codes: J04AA, J04AB, J04AC, J04AD, J04AK, and J04AM)

**Statin drugs (HMG CoA reductase inhibitors):** simvastatin, lovastatin, pravastatin, fluvastatin, atorvastatin, cerivastatin, rosuvastatin, pitavastatin (ATC code: C10AA, C10BA, and C10BX)

**Corticosteroid drugs:** betamethasone, dexamethasone, fluocortolone, methylprednisolone, paramethasone, prednisolone, prednisone, triamcinolone , hydrocortisone, cortisone (ATC code: H02AB and H02B)

**E-table 1.** Summary of tuberculosis (TB) contact investigation strategy in Taiwan

| **Timing and Survey for Contacts** | | **Characteristics of Index Case** | | |
| --- | --- | --- | --- | --- |
| Timing after index case diagnosis | Survey | Open TB* or cavitations on CXR | Not open TB and no cavitations on CXR | Extra-pulmonary TB |
| Within 1 month | CXR for those ≥13 y/o | ✔ | ✔ | ✔ |
|  | CXR and TST for <13 y/o | ✔ | ✔ | ✔ |
| At 3^rd^ month | 2^nd^ TST for <13 y/o with negative TST at 1^st^ month,  and 1^st^ TST for contacts born after 1986 since 2012 | ✔ | ✘ | ✘ |
| At 1 year | Second CXR | ✔ | ✘ | ✘ |

**Note.** CXR, chest X-ray; TST, tuberculin skin test

* Open TB was defined as sputum culture-positive for *Mycobacterium tuberculosis* complex and/or smear-positive for acid-fast bacilli.

**E-Table 2.** Factors associated with metformin use in logistic regression analysis while performing propensity-score matching

| Factors | Adjusted OR (95% CI) | *p*-value |
| --- | --- | --- |
| Male vs. female | 0.89 (0.83–0.95) | <0.001 |
| Age at index date ≥65 vs. <65 years | 0.71 (0.67–0.77) | <0.001 |
| aDCSI score ≥1 vs. <1 | 1.21 (1.12–1.31) | <0.001 |
| Type 1 DM | 0.48 (0.38–0.62) | <0.001 |
| Liver cirrhosis | 0.51 (0.31–0.81) | 0.005 |

Abbreviations: aDCSI, adapted Diabetes Complications Severity Index; DM, diabetes mellitus; OR, odds ratio.

**E-Table 3.** Clinical characteristics of metformin users and non-users before matching

| Characteristics | Met. nonusers  (n=9,289) | Met. users  (n=5,847) | *p*-value^#^ |
| --- | --- | --- | --- |
| Age at index date |  |  | <0.001 |
| <45 | 863 (9.3%) | 531 (9.1%) |  |
| 45 to 65 | 4,266 (45.9%) | 3,067 (52.5%) |  |
| ≥65 | 4,160 (44.8%) | 2,249 (38.5%) |  |
| Sex |  |  | 0.024 |
| Male | 4089 (44.0%) | 2465 (42.2%) |  |
| Female | 5200 (56.0%) | 3382 (57.8%) |  |
| aDCSI score |  |  | <0.001 |
| ≥3 | 3333 (35.9%) | 2010 (33.4%) |  |
| 1 to 2 | 3339 (35.9%) | 2294 (39.2%) |  |
| 0 | 2617 (28.2%) | 1543 (26.4%) |  |
| Type 1 DM | 258 (2.8%) | 88 (1.5%) | <0.001 |
| Liver cirrhosis | 71 (0.8%) | 23 (0.4%) | 0.005 |
| Coexisting medical condition |  |  |  |
| Previous TB history | 392 (4.2%) | 224 (3.8%) | 0.238 |
| COPD | 785 (8.5%) | 352 (6.0%) | <0.001 |
| Malignancy | 479 (5.2%) | 234 (4.0%) | 0.001 |
| Bronchiectasis | 175 (1.9%) | 64 (1.1%) | <0.001 |
| Transplantation | 5 (0.1%) | 5 (0.09%) | 0.460 |
| HIV/AIDS | 5 (0.1%) | 2 (0.05%) | 0.585 |
| Other co-morbidity^$^ | 183 (2.0%) | 96 (1.6%) | 0.144 |
| Urban contact area | 6269 (67.5%) | 4035 (69.0%) | 0.051 |
| Local TB incidence (/100,000 PYs) | 58.05 ± 15.92 | 59.08 ± 15.72 | <0.001 |
| Low income | 641 (6.9%) | 426 (7.3%) | 0.367 |
| Medical visits in 3 months | 3.78 ± 3.07 | 4.02 ± 2.92 | <0.001 |
| Statin users | 1637 (17.6%) | 1559 (26.7%) | <0.001 |
| Corticosteroid users | 123 (1.3%) | 67 (1.1%) | 0.338 |
| Insulin users | 3789 (40.8%) | 2399 (41.0%) | 0.771 |
| Other OHA users | 8206 (88.3%) | 5552 (95.0%) | <0.001 |
| Latent TB infection | 391 (4.2%) | 200 (3.4%) | 0.015 |
| IPT | 94 (1.0%) | 65 (1.1%) | 0.558 |
|  |  |  |  |
| Follow-up duration (days) | 529.00 ± 255.58 | 637.32 ± 166.05 | <0.001 |
| Incident TB events | 117 (1.3%) | 77 (1.3%) | 0.760 |

Abbreviations: aDCSI, adapted Diabetes Complications Severity Index; HIV/AIDS, human immunodeficiency virus/acquired immunodeficiency syndrome; COPD, chronic obstructive pulmonary disease; DM, diabetes mellitus; IPT, early adherent isoniazid preventive therapy; Met., metformin; OHA, oral hypoglycemic agents; PY, person-years; TB, tuberculosis.

Data are expressed as the number (%) unless otherwise specified.

^#^ *p* value of metformin users *vs.* nonusers in independent *t* test for continuous variables and chi square test for categorical variables.

^$^ Including pneumoconiosis, psoriasis, rheumatoid arthritis, and ankylosing spondylitis.

**E-Table 4.** Cumulative defined daily doses (DDDs) of metformin between metformin user and nonuser within two years after index date

|  | First year | |  | Second year | |
| --- | --- | --- | --- | --- | --- |
|  | Mean + SD | Median [IQR] |  | Mean + SD | Median [IQR] |
| Metformin user | 215.2 ± 191.8 | 183.9 [42.0, 319.2] |  | 143.4 ± 187.7 | 43.4 [0, 265.8] |
| Metformin nonuser | 45.4 ± 84.7 | 0 [0, 60.4] |  | 47.3 ± 96.4 | 0 [0, 45.8] |

Abbreviation: IQR, interquartile range; SD, standard deviation;

**E-Table 5.** Crude hazards ratio for active tuberculosis among patients with diabetes after propensity score matching for metformin use, as determined through univariate stratified cox proportional hazard regression analysis

| Predictors | crude HR (95% CI) | *p*-value |
| --- | --- | --- |
| DM status |  |  |
| DM, metformin nonusers | Reference |  |
| DM, metformin users | 0.69 (0.51 – 0.92) | 0.012 |
| Healthy cohort | 0.44 (0.32 – 0.62) | <0.001 |
| Age at index date |  |  |
| <65 | Reference |  |
| ≥65 | 1.48 (1.15 – 1.90) | 0.002 |
| Sex |  |  |
| Female | Reference |  |
| Male | 2.76 (2.11 – 3.60) | <0.001 |
| aDCSI score |  |  |
| 0 | Reference |  |
| 1-2 | 1.98 (1.29 – 3.02) | 0.002 |
| ≥3 | 1.75 (1.16 – 2.64) | 0.008 |
| Type 1 DM | 38.41 (0.03 – 46747.31) | 0.314 |
| Coexisting medical condition |  |  |
| Previous TB history | 1.06 (0.54 – 2.07) | 0.864 |
| COPD | 1.10 (0.60 – 2.04) | 0.751 |
| Malignancy | 1.26 (0.50 – 3.19) | 0.627 |
| Bronchiectasis | 12.00 (1.45 – 99.67) | 0.021 |
| Transplantation | 169.48 (0.000002 – 14363457666.62) | 0.582 |
| HIV/AIDS | 0.02 (0.000000002 – 147346.84) | 0.610 |
| Liver cirrhosis | 38.41 (0.0002 – 8479604.92) | 0.561 |
| Other co-morbidity^$^ | 0.56 (0.15 – 2.06) | 0.386 |
| Urban contact area | 0.86 (0.62 – 1.21) | 0.390 |
| Local TB incidence (/100,000 PYs) | 1.01 (0.99 – 1.01) | 0.306 |
| Low income | 0.97 (0.51 – 1.83) | 0.914 |
| Medical visits in 3 months | 1.06 (1.01 – 1.11) | 0.024 |
| Statin users | 0.81 (0.50 – 1.29) | 0.372 |
| Corticosteroid users | 5.33 (1.42 – 20.10) | 0.013 |
| Insulin users | 1.82 (1.32 – 2.52) | <0.001 |
| Other OHA users | 1.78 (1.31 – 2.42) | <0.001 |
| Latent TB infection | 1.18 (0.60 – 2.35) | 0.634 |
| IPT | 1.00 (0.23 – 4.35) | >0.999 |

Abbreviations: aDCSI, adapted Diabetes Complications Severity Index; COPD, chronic obstructive pulmonary disease; DM, diabetes mellitus; HIV/AIDS, human immunodeficiency virus/acquired immunodeficiency syndrome; IPT, early adherent isoniazid preventive therapy; OHA, oral hypoglycemic agents.

^$^Including pneumoconiosis, psoriasis, rheumatoid arthritis, and ankylosing spondylitis.
